# The support needs of terminally ill people living alone at home: a narrative review

**DOI:** 10.1080/21642850.2014.933342

**Published:** 2014-09-25

**Authors:** Samar M. Aoun, Lauren J. Breen, Denise Howting

**Affiliations:** ^a^ School of Nursing and Midwifery, Curtin University, GPOBox U1987, Perth6845, Australia; ^b^ School of Psychology and Speech Pathology, Curtin University, Perth, Australia

**Keywords:** palliative care, home care, living alone, hospice, social support, place of death

## Abstract

*Context*: The number of terminally ill people who live alone at home and without a caregiver is growing and exerting pressure on the stretched resources of home-based palliative care services. *Objectives*: We aimed to highlight the unmet support needs of terminally ill people who live alone at home and have no primary caregiver and identify specific models of care that have been used to address these gaps. *Methods*: We conducted a narrative review of empirical research published in peer-reviewed journals in English using a systematic approach, searching databases 2002–2013. This review identified 547 abstracts as being potentially relevant. Of these, 95 were retrieved and assessed, with 37 studies finally reviewed. *Results*: Majority of the studies highlighted the reduced likelihood of this group to be cared for and die at home and the experiences of more psychosocial distress and more hospital admissions than people with a primary caregiver. Few studies reported on the development of models of care but showed that the challenges faced by this group may be mitigated by interventions tailored to meet their specific needs. *Conclusion*: This is the first review to highlight the growing challenges facing community palliative care services in supporting the increasing number of people living alone who require care. There is a need for more studies to examine the effectiveness of informal support networks and suitable models of care and to provide directions that will inform service planning for this growing and challenging group.

## Introduction and background

The fundamental aim of palliative care is to achieve the best quality of life possible and support for terminally ill people and their families (World Health Organization, 2007). (National Hospice and Palliative Care Organization, 2008; National Institute for Health and Clinical Excellence, 2004; Palliative Care Australia, 2005). Family or informal caregivers provide unpaid help and support to a relative, friend, or neighbor receiving palliative care who could not manage otherwise because of frailty, illness, or disability. These caregivers commonly play a central role in home-based palliative care undertaking tasks such as symptom assessment and management, personal care, and administering medications (Aoun, Kristjanson, Currow, & Hudson, 2005; Aoun, Kristjanson, Hudson, Currow, & Rosenberg, 2005; Funk et al., 2010; Hauser & Kramer, 2004; Stajduhar et al., 2010). In so doing, they provide much of the support needed by the patient as well as reducing the costs of formal care (Aoun, Kristjanson, Currow, et al., 2005; Aoun, Kristjanson, Hudson, et al., 2005; Breen, 2012; Haines, 2011).

Without the contribution of family or informal caregivers, the well-being of most terminally ill people would be compromised (Aoun, Kristjanson, Hudson, et al., 2005; Hudson, 2003). Although approximately one-third of all Australian patients receiving palliative care services die at home, up to 90% of terminally ill patients spend the majority of their last year of life at home (Australian Institute of Health and Welfare, 2012). In a systematic review of variables affecting place of death for terminally ill cancer patients in the UK, living with relatives was identified as strongly associated with dying at home (Gomes & Higginson, 2006). Several reports have argued that the changing demographics of end of life, including the reduction in numbers of potential family caregivers, have considerable outcomes for service delivery (Hudson, 2003; Rolls, Seymour, Froggatt, & Hanratty, 2011; Turis, 2006).

### Aging population and informal caring needs

Factors such as an aging population, declining fertility rate, and the rising employment of women are reducing the availability of informal caregivers (Australian Institute of Health and Welfare, 2007). Living alone increases with age; currently, 29% of Australians aged 65 years and over, and 39% of those aged 85 years and over, live alone (Australian Bureau of Statistics, 2012). In Australia, the population share of those aged 65 years and over is projected to double from 13% to 27%, while the share of those 80 years and over will more than triple from 3% to 11% between 2002 and 2031 (National Centre for Social and Economic Modelling, 2004). Statistics are similar in the UK (31% over 65 years living alone) (Office for National Statistics (UK), 2013) and Canada (25% over 65 years living alone) (Statistics Canada, 2012). A multisite survey of palliative care centers servicing over 3000 patients in 21 European countries showed that 28% of patients lived alone (Kaasa, Torvik, Cherny, Hanks, & de Conno, 2007). Predictions for demand on services in the UK point to a 67% increase in the number of people with disability requiring care by 2025 (Jagger et al., 2006).

These figures draw attention to the growing group of persons who are likely to be without an informal caregiver, as their needs for care increase. It is likely that the demand for informal care will outstrip supply over the coming years. For instance, in Australia in 2001, 43% of people aged 65 years and over living in private dwellings and needing care did not have an informal caregiver and this proportion is projected to increase to 65% by 2031 (National Centre for Social and Economic Modelling, 2004). Thus, there may be a greater demand for institutional care due to an inadequate supply of informal caregivers. However, as many older people are likely to prefer options that support and allow them to stay in their own homes, there will be ongoing pressure on community-based support.

### Definition of living alone and profile of those receiving palliative care

According to statistical reports (Office for National Statistics (UK), 2013), an individual who lives alone represents a one-person household and no one else shares this address with them. By contrast, having a single status does not mean that they do not live with other people. The profile of terminally ill people living alone can be gleaned mainly from studies conducted by Aoun and colleagues in Australia (Aoun et al., 2007; Aoun, O'Connor, Skett, Deas, & Smith, 2012), where researchers analyzed 721 records from 3 large Australian home-based palliative care services. Home alone clients made up between 7% and 12% of the total caseload of these services: 47% were male, mean age at death was 75 years, and the majority had cancer with only 3.1% with non-cancer diagnoses. The clients had been living alone at home for a median of 13 years, ranging from less than a year to 60 years. Nearly 80% of the participants reported that they were living alone by choice rather than by circumstance.

### Challenges in provision and access to care

The proportion of patients supported at home until death, if this is their preference, remains an important performance indicator for many community palliative care programs (Brogaard, Neergaard, Sokolowski, Olesen, & Jensen, 2013). A large survey of 9344 people residing in several European countries reported that 68.2% of participants indicated a preference to die at home if they were faced with terminal cancer (Gomes et al., 2012). A similar study in Canada also showed that 70.8% of 1203 respondents preferred to be at home at end of life (Wilson, Cohen, Deliens, Hewitt, & Houttekier, 2013). Focus groups and interviews with adults (65 years of age and older) in the UK showed that, despite home being the preferred place of care, the presence of informal caregivers was cited as a mediator to achieving this outcome (Gott, Seymour, Bellamy, Clark, & Ahmedzai, 2004).

Dying at home is also the stated preference of many people receiving palliative care, at least in the earlier stages of their final illness. A recent systematic review of the literature on preferences for places of death showed that most adults at end of life indicate a preference to die at home, and most do not change their preference during disease progression (Gomes, Calanzani, Gysels, Hall, & Higginson, 2013). A study of Danish cancer patients demonstrated that 84% preferred to be cared for at home and 71% preferred to die at home (Brogaard et al., 2013).

However, terminally ill people are far more likely to die in a hospital or a similar facility rather than at home. A study of all cancer deaths in England from 1993 to2010 showed that hospital was the most common place of death (48%), followed by home (24.5%) and hospice (16.4%) (Gao, Ho, Verne, Glickman, & Higginson, 2013). Similarly, a study in Spain showed that only 17% of cancer patients died at home; the majority (74%) died in hospital and the remainder died in either a hospice (6%) or a nursing home (3%) (Alonso-Babarro et al., 2013). However, home-based palliative care programs may assist people to stay at home for longer and to die at home, if this is their wish. For example, people receiving home-based hospice care in Taiwan were more likely to die at home (60.8%) than in a hospital (39.2%) (Lee, Hu, Loh, & Hwang, 2013).

Despite these preferences, facilitating place of death wishes is a challenge for services. A postal survey of Australian health professionals working in home-based palliative care (Aoun et al., 2007) demonstrated that more than 60% reported spending at least 15–30 minutes of additional time per visit to support clients without a caregiver with symptom control, medications, mobility, transport, and social support. Furthermore, nearly half (43%) reported spending one hour or more of additional time per visit on activities of daily living, while 25% reported taking one hour or more of additional time per visit for symptom control, housekeeping, and emotional support. The nurses reported concerns such as limited staffing, lack of a social worker and housekeeping support, and client safety. Postal surveys and interviews with Australian service providers regarding their perspectives on resources needed to look after home alone dying clients revealed that, while service providers expressed a respect for the clients' autonomy and independence, they felt pressured to ensure that safe and attentive care was possible. They identified the inability of home alone clients to anticipate their needs should their condition deteriorate, and they struggled to make them understand the limitations of their situation (Aoun, Wall, Kristjanson, & Shahid, 2013).

Given the aging population, the growing reliance on informal carers, and the preference to be cared for and die at home, it is likely that home-based palliative care services are facing increasing challenges in servicing the needs of such clients. However, there is limited evidence base upon which service providers may draw in order to inform service planning for a growing population.

## Objectives

The objectives of the review were to

1. highlight the unmet support needs of terminally ill people who live alone at home and have no primary caregiver and
2. identify specific models of care that have been used to address these gaps.

## Methodology

**Figure 1.**
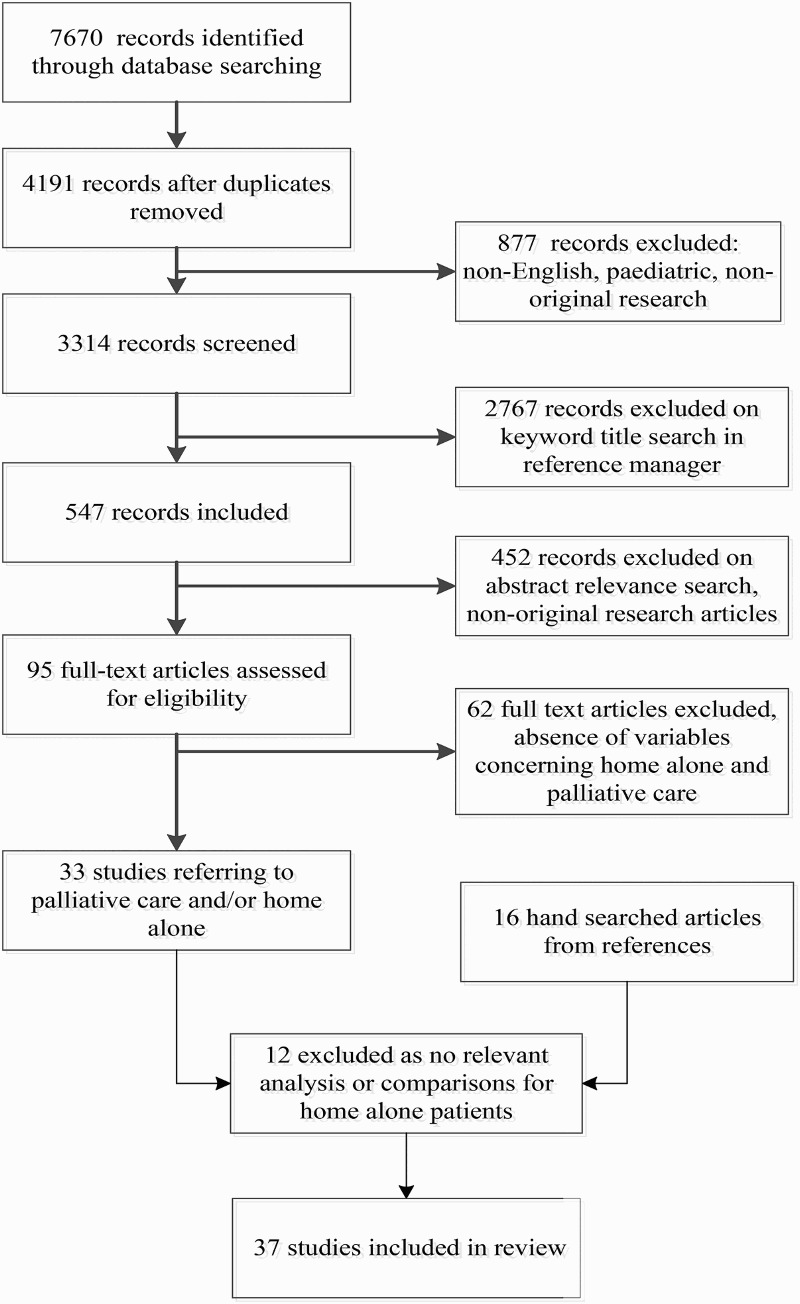
Flow chart showing articles that were identified, screened, eligible, and included in the review.

Using the Preferred Reporting Items for Systematic Reviews and Meta-Analyses (PRISMA) statement as a guide, a literature search was undertaken of the following databases: Ovid MEDLINE and EMBASE, ProQuest Medline, NLM MEDLINE, PyscINFO, CINAHL, and CareSearch. We considered national and international literature published, or available online in 2013, in refereed journals in the last 11 years (January 2002–October 2013). We included only articles published in English. Searches were undertaken using the following key words: palliative care, hospice care, end of life, supportive care, life-limiting illness and terminal illness, models of care, and interventions, in conjunction with associate keywords: home alone, living alone, no carer/caregiver, home care, place of care, and place of death. Figure 1 shows the search process and outcomes.

We conducted a comprehensive narrative review of empirical research on the support needs of terminally ill people living alone at home, with a systematic approach that utilized several clear inclusion/exclusion criteria. Exclusions were not made based upon assessments of the rigor, validity, and reliability of studies and their findings, as would be typical in a systematic review. All three authors participated in the coding of the identified articles. We took a directed content analysis approach (Hsieh & Shannon, 2005) in order to group the articles into themes relating to either unmet support needs or models of care. We discussed all differences and achieved consensus, indicating the trustworthiness of the coding scheme (Mays & Pope, 2000). Our goal was to provide a broad, comprehensive overview of published research in this field, as opposed to a systematic review that focuses on generating a definitive answer to a narrowly defined research question.

All search results were downloaded into a reference manager (EndNote X5, Thomson Reuters). This initial search elicited 547 records for further review. After deletion of duplicate records, we removed articles that were not available in English, involved pediatrics, or were not obvious original research. Preliminary screening to exclude non-relevant articles and further non-original research articles led to the exclusion of 452 papers, resulting in the retention of 95 full articles for eligibility assessment for inclusion in the review. We excluded 62 articles due to the absence of variables concerning home alone and palliative care. A further hand search of the relevant references of these articles was undertaken to capture articles that might have been missed on the initial search. Initially, 49 studies met the inclusion criteria for our review. However, 12 studies were later excluded because, although they described samples wherein a considerable proportion of their study population was living alone, the authors provided no comparisons or analyses relevant to places of care and death or physical and psychosocial health and well-being. Therefore, 37 studies are included in the results section.

## Results

This section describes the unmet support needs of terminally ill people who live alone at home in terms of their places of care and death, and also their physical and psychosocial well-being and identifies specific models of care that have been used to address these gaps.

### The unmet needs of terminally ill people living alone

Thirty-five (95%) of the included studies described the unmet needs of terminally people who live alone. Although different methodologies were used in these studies, ranging from prospective designs to retrospective review of health data, the proportion of people living alone in their study population was considerable. Our review of these studies revealed two main disadvantages concerning the unmet needs of terminally people who live alone: places of care and death (Table 1) and physical and psychosocial well-being (Table 2).

**Table 1.** Studies reporting on place of death outcomes for terminally ill people living alone in their study population.

Study	Country	Population	Design	Living alone^a^	Outcomes
(1) Aabom, Kragstrup, Vondeling, Bakketeig, and Stovring (2005)	Denmark	Deceased adult cancer patients	Retrospective study of all cancer deaths over three years (1996–1998) (*n* = 4386)	Not stated; inferred by absence of a spouse	Death at home was significantly less likely for people who were widowed, divorced, or single at the time of death
(2) Ahlner-Elmqvist, Jordhøy, Jannert, Fayers, and Kaasa (2004)	Sweden	Adult palliative cancer patients	Prospective, non-randomized study of patients who received either advanced home care (*n* = 119) or conventional hospital care (*n* = 178)	25% of advanced home care group and 32% of conventional hospital care group	Irrespective of intervention condition, death at home was significantly less likely for patients who lived alone
(3) Alonso-Babarro et al. (2011)	Spain	Deceased adult cancer patients	Retrospective study of cancer deaths in two metropolitan areas (*n* = 524)	Not stated; inferred by absence of a spouse	Death at home was significantly less likely for people who were separated, divorced, or single at the time of death
(4) Aoun and Skett (2013)	Australia	Adult palliative care patients	Longitudinal study of congruence between preference for and place of death	100%	While half expressed a preference to die at home, only 14% died at home
(5) Aoun et al. (2007)	Australia	Adult palliative care patient records and surveys of health professionals	Retrospective study of home-based palliative care recipients over 15 months (*n* = 721)	100%	Compared to patients with caregivers, patients living alone were less likely to die at home and more likely to stay in hospital for longer
(6) Aoun, Kristjanson, Oldham, and Currow (2008)	Australia	Adult palliative care patients	Interviews with home-based palliative care recipients (*n* = 11)	100%	Participants preferred to be at home at the end of their life; however, they were unable to describe the types of support required to help them remain at home
(7) Brazil et al. (2002)	Canada	Adult caregivers of people who died approximately nine months earlier	Retrospective cohort study using telephone interviews (*n* = 151)	14% of patients lived alone but all had caregivers	Death was significantly more likely to occur at home when the patient lived with the caregiver
(8) Brink and Smith (2008)	Canada	Adults receiving palliative home care	Retrospective review of palliative home care patients over 27 months (2004–2006) (*n* = 536)	22%	Patients who lived alone were significantly less likely to die at home alone
(9) Brogaard et al. (2013)	Denmark	Adults with advanced cancer	Prospective study of terminal cancer patients using interviews and questionnaires (*n* = 96)	Not stated; inferred by absence of a spouse	Home death was significantly more likely for those who lived with their partner
(10) Carlsson and Rollison (2003)	Sweden	Adult caregivers of people receiving palliative home care	Retrospective review of death of patients receiving palliative home care over one year (*n* = 180)	40% of patients lived alone but all had caregivers	Home death was significantly less likely for those who lived alone
(11) Cohen et al. (2006)	Belgium	Residents ≥1 year of age who died	Retrospective study of death certificate data and health care statistics for all deaths (2001) (*n* = 55,759)	18%	Patients living alone were significantly less likely to die at home
(12) Cohen et al. (2010)	Italy, Belgium, Netherlands, Norway, and UK	Deceased cancer patients	Study of death certificate data and health care statistics for all cancer deaths (2002–2003) (*n* = 238,216)	Not stated; inferred by absence of a spouse	Patients who were married at the time of death were significantly more likely to die at home than those who were unmarried, divorced, or widowed
(13) Gao et al. (2013)	UK	Deceased cancer patients	Retrospective analysis of all cancer deaths in England between 1993 and 2010 (*n* = 2,281,223)	Not stated; inferred by absence of a spouse	Death at home was significantly less likely for people who were single, widowed, or divorced at the time of death
(14) Goodridge, Lawson, Rennie, and Marciniuk (2010)	Canada	Rural residents with advanced respiratory disease in the last 12 months of life	Retrospective cohort of patients who died in 2004 of lung cancer or COPD (*n* = 1098)	Not stated; inferred by absence of a spouse	Hospital death was significantly less likely for people who were never married
(15) Grundy, Mayer, Young, and Sloggett (2004)	UK	Adults living with cancer	Prospective nationally representative data linkage study (1991–1995) (*n* = 6257)	28%	Patients living alone were significantly less likely to die at home
(16) Gyllenhammar et al. (2003)	Sweden	Palliative cancer patient deaths	Prospective study of palliative cancer patient deaths in 1999 (*n* = 221)	24%	Living with a spouse or other family member was significantly associated with dying at home
(17) Houttekier et al. (2009)	Belgium	Residents who died from chronic diseases eligible for palliative care	Retrospective study of death certificate data and health care statistics for all deaths (2003) (*n* = 3672)	Not stated; inferred by absence of a spouse	Patients who were married at the time of death were significantly more likely to die at home
(18) Jakobsson, Johnsson, Persson, and Gaston-Johansson (2006)	Sweden	Adults deaths in 2001 (excluding accidents, suicide, and sudden deaths)	Retrospective study of death certificates and health records of a random sample of deaths in 2001 (*n* = 229)	59%	People who did not live with a partner at end of life were significantly more likely to die in residential homes than those who resided with their partner
(19) Jayaraman and Joseph (2013)	Canada	Adults deaths (≥19 years of age) from 2004 to 2008 in British Columbia	Retrospective study of all death records 2004–2008 (*n* = 153,111)	Not stated; inferred by absence of a spouse	Patients who were married at the time of death were significantly more likely to die at home.
(20) Jordhøy, Saltvedt, and Fayers (2003)	Norway	Deceased adult cancer patients in a trial of palliative care	Prospective study of deaths of people who entered a palliative care program (1995–1997) (*n* = 395)	32%	A significant difference between place of death according to living arrangements, with those who lived alone being significantly less likely to die at home than those who lived with a spouse
(21) Lee et al. (2013)	Taiwan	Patients with cancer receiving hospice home care	Retrospective study of hospice home care cancer patients over three years, 2009–2011 (*n* = 439)	Not stated; inferred by absence of a spouse	Death at home was significantly less likely for people who were single or divorced at the time of death
(22) Masucci, Guerriere, Cheng, and Coyte (2010)	Canada	Caregivers of palliative care patients with cancer	Prospective study using fortnightly telephone interviews (2005–2007) (*n* = 110)	10% of patients lived alone but all had caregivers	Those living alone were significantly less likely to die at home than those who lived with their caregiver
(23) Neergaard et al. (2009)	Denmark	GPs of deceased adult cancer patients	Retrospective study of cancer patient deaths in 2006 (*n* = 333)	Not stated; 40% of patients were single and 60% were married	No significant difference in likelihood of home death between single and married patients
(24) Pinzón et al. (2011)	Germany	Relatives of deceased	Retrospective survey of relatives for deaths in 2005 (*n* = 1378)	Not stated; inferred by absence of a spouse	There was a significant association between marital status and place of death, with home death more likely for married than non-married people
(25) Tang (2003)	USA	Adults (21 years and older) with terminal cancer	Interviews with terminally ill cancer clients (*n* = 180)	Not stated	Almost all expressed a preference to die at home, but those who lived alone felt unable to enact their desire due to a lack of caregiver support
(26) Tang and McCorkle (2003)	USA	Adults (21 years and older) with terminal cancer	Prospective cohort study of terminally ill cancer patients (*n* = 180)	26.8%	While almost all preferred to die at home, those who lived alone felt unable to due to a lack of caregivers
(27) Taylor, Ensor, and Stanley (2012)	New Zealand	Deceased hospice patients	Retrospective review of patient charts of all patients in one hospice who died in 2006–2008 (*n* = 1268)	Not stated; inferred by absence of a spouse	Unmarried patients were significantly more likely to die in an aged care or residential facility than at home
(28) Tiernan, O'Connor, O'Siorain, and Kearney (2002)	Ireland	Home-based palliative care patients	Prospective study of patients' preferences for place of death, actual place of death, and health records (*n* = 191)	10%	Patients who lived alone were significantly less likely to die at home than patients who did not live alone

Note: COPD, chronic obstructive pulmonary disease.

^a^Rounded to nearest whole number.

**Table 2.** Studies reporting on physical and psychosocial well-being outcomes for terminally ill people living alone in their study population.

Study	Country	Population	Design	Living alone^a^	Outcomes
(1) Aoun et al. (2007)	Australia	Adult palliative care patients	Retrospective study of home-based palliative care recipients’ records over 15 months (*n* = 721)	100%	Compared to patients with caregivers, patients who lived alone were likely to have service delivery for longer but received fewer home visits, to require more assistance with activities of daily living but receive less equipment, oxygen, and counselling
(2) Aoun et al. (2008)	Australia	Adults living alone with a terminal illness	Semi-structured interviews (*n* = 11)	100%	Participants described their challenges in addressing their physical, social, emotional, and existential needs
(3) Chibnall, Videen, Duckro, and Miller (2002)	USA	Adults living with life-threatening illnesses	Cross-sectional data comprising a series of measures (*n* = 67)	34%	Living alone was significantly associated with higher death distress
(4). Currow et al. (2008)	Australia	Terminally ill people accessing home-based palliative care	Retrospective study of home-based palliative care service data over three years (2003–2006) (*n* = 5,203)	10%	Those who lived alone were significantly less likely to access oxygen treatment and had had twice as many visits by clinicians before being prescribed oxygen
(5). Goodridge et al. (2010)	Canada	Rural residents with advanced respiratory disease in the last 12 months of life	Retrospective cohort of patients who died in 2004 of lung cancer or COPD (*n* = 1098)	Not stated; inferred by absence of a spouse	Widows/widowers had significantly fewer physician visits in the 12 months prior to death; people who had never married or were separated/divorced had significantly fewer hospitalizations
(6) Hanratty et al. (2013)	UK	Adult palliative care patients expected to die within 12 months	In-depth qualitative interviews (*n* = 32)	62.5%	The home alone participants described being disadvantaged in terms of access to practical and emotional supports and their ability to direct their care
(7) Iliffe et al. (2007)	UK	Adults (65 years and older) living without disabilities in the community	Retrospective analysis of baseline data from an RCT of health risks in older people (*n* = 2641)	33%	Living alone was significantly associated with risk of social isolation and depressed mood
(8). Johnson, Gallagher, and Wolinsky (2004)	USA	Adults (70 years and older) living in the community who rely on in-home informal care	Longitudinal study with four data collection points (1984, 1986, 1888, and 1990) (*n* = 7527)	Not stated	Older adults who lived alone reported receiving significantly less assistance with activities of daily living than those who lived with others or were married
(9) Kharicha et al. (2007)	UK	Adults (65 years and older) living without disabilities in the community	Retrospective analysis of baseline data from an RCT of health risks in older people (*n* = 2641)	33%	People living alone were significantly more likely to report a range of psychosocial difficulties in relation to activities of daily living, mood, memory, physical activity, vision, diet, alcohol use, multiple falls, and social isolation

^a^Rounded to nearest whole number.

#### Places of care and death

Several studies (*n* = 28, 76%) provided data on places of care and death. Irrespective of methodology and country of origin, these studies demonstrated that people were less likely to die at home when they lived alone (Table 1). In several studies, people living alone were described briefly as a subgroup of the total study sample of people receiving palliative care having a decreased likelihood of a home death (Ahlner-Elmqvist et al., 2004; Brink & Frise Smith, 2008; Cohen et al., 2006; Grundy et al., 2004; Gyllenhammar et al., 2003; Jordhøy et al., 2003; Tang & McCorkle, 2003; Tiernan et al., 2002). These studies demonstrating disadvantage in place of care and death are complemented by subjective data. For instance, interviews with terminally ill patients who live alone in the USA (Tang, 2003; Tang & McCorkle, 2003) and Australia (Aoun et al., 2008) revealed that, while most preferred to die at home, some reported feeling unable to do so due to limited supports. These perceptions were corroborated in a longitudinal study of end-of-life preferences of Australian home alone clients who demonstrated a preference to die at home (49%) over dying in a hospice (23%) or a hospital (12%); 16% indicated no preference (Aoun & Skett, 2013). However, only 14% died at home, while 56% died in a hospice and 22% in a hospital. Overall, congruence between preferred and actual place of death decreased from 53% to 41% during the course of the study, possibly due to clients growing more confident of achieving a home death after using home-based care for a period of time.

Additionally, the design of some studies rendered this group overlooked, for instance, by sampling caregivers to determine variables associated with home death (Brazil, Bedard, & Willison, 2002; Carlsson & Rollison, 2003; Masucci et al., 2010) or by not reporting home alone status and/or inferring it from the absence of a caregiver or being single (Aabom et al., 2005; Alonso-Babarro et al., 2013; Brogaard et al., 2013; Cohen et al., 2010; Gao et al., 2013; Goodridge et al., 2010; Houttekier et al., 2009; Jakobsson et al., 2006; Jayaraman & Joseph, 2013; Lee et al., 2013; Neergaard et al., 2009; Pinzón et al., 2011; Tang, 2003; Taylor et al., 2012). Only one study (3%) focused on the place of death outcomes of this subgroup and reported that, compared to patients with a caregiver, those without a caregiver were less likely to die at home (35% compared to 57%), twice as likely to die in a hospice, and 2.5 times as likely to die in a hospital (Aoun et al., 2007).

#### Physical and psychosocial well-being

Nine studies (24%) highlighted the considerable health and psychosocial disadvantages experienced by people living alone at end of life (Table 2; NB: Two of these studies also provided data on places of care and death and therefore appear in both tables). People living alone at the end of life reported more distress, poorer adjustment to diagnosis, and reduced quality of life, and received less help with activities of daily living than those living with others (Chibnall et al., 2002; Johnson et al., 2004). Interviews with adult palliative care clients living at home without a caregiver in the UK (Hanratty et al., 2013) and Australia (Aoun et al., 2008) showed that patients faced challenges in meeting their care needs and perceived they were disadvantaged in terms of receipt of practical and emotional supports.

People living alone at end of life were also more likely to experience a range of problems with falls, diet, smoking, social isolation, and chronic health conditions (Iliffe et al., 2007; Kharicha et al., 2007). Notwithstanding these additional struggles, they received half as many home visits, despite being enrolled in the service for an average of 20 days longer, and were more likely to be admitted to hospital. They required more equipment, support with hygiene, home help, and liaison with other health professionals (Aoun et al., 2007; Currow et al., 2008; Goodridge et al., 2010). Thus, these studies showed that the presence of a caregiver in the home was related to better physical and psychosocial well-being compared to those without a caregiver and more timely and improved access to required treatments at end of life.

### Models of care for terminally ill people living alone

Two studies (5%) explored models of care for terminally ill people who live alone (Table 3). These models of care were based upon formative data provided by three studies – one focusing on the perspectives of clients (Aoun et al., 2008) and two on service providers (Aoun et al., 2007; Aoun, Wall et al., 2013). The first study was a pilot intervention using a randomized trial design to test two models of care (personal alarms and additional care-aide support) compared to standard care (Aoun et al., 2013). The findings indicated that those who received care-aide support tended to have improved scores (lower scores) in appetite problems and fatigue compared to the other two groups. The second study sourced qualitative feedback from these participants on the benefits and barriers of these interventions (Aoun et al., 2012). The care-aide model of care resulted in benefits such as easing the burden of everyday living, supporting well-being, preserving a sense of dignity, and reducing loneliness and isolation, while the personal alarm model of care imparted a sense of security, provided peace of mind, and helped clients manage feelings of isolation. Importantly, participants in both groups felt they could remain at home longer. By providing a safer, more secure environment through the use of either model of care, clients were able to continue their activities of daily living, and could build a sense of ‘normality’ into their lives with a degree of independence through support and dignity. Together, these two studies showed that the challenges faced by terminally ill people who live alone may be mitigated by interventions tailored to meet their specific needs.

**Table 3.** Studies reporting on models of care for terminally ill people living alone.

Study	Country	Population	Design	Outcomes
(1) Aoun et al. (2012)	Australia	Adults living alone with a terminal illness	In-depth qualitative study using face-to-face semi-structured interviews (*n* = 26) exploring the use of personal alarm or additional care-aide support	Personal alarms provided a sense of security and reduced feelings of isolation; additional care-aide time assisted with activities of daily living, enhanced quality of life, maintained dignity, and reduced feelings of isolation
(2) Aoun, O'Connor, Breen, Deas, and Skett (2013)	Australia	Adults living alone with a terminal illness	RCT of personal alarm or additional care-aide support in comparison to standard care	Care-aide support improved sleeping and appetite

## Discussion

This review has described the unmet needs of terminally ill people living alone, the disadvantages they face in terms of their physical and psychosocial health and well-being, and their reduced likelihood to be cared for and die at home. It also reported on potential management options to enable this group to remain at home for as long as possible. Additionally, this review has highlighted three factors that would intensify the challenges of providing community and home-based palliative care services: (1) an increase in the number of people living alone who require care; (2) a decrease in the provision and availability of family caregivers; and (3) people's preference to be supported to die in their own homes.

For people living with a terminal illness, the presence of a caregiver and family support proves to be the strongest independent factor associated with home death (Gomes & Higginson, 2006). Living alone raises both the probability and the necessity for living and being cared for in institutions (Rolls et al., 2011), which may be less preferable from a patient perspective and more expensive as home care tends to be more cost-effective than hospital care (Higginson, Jarman, Astin, & Dolan, 1999). Furthermore, hospitals are not recognized as ideal places for terminal care due to a number of problems including communication difficulties between hospital staff and patient/family, being overlooked by staff on wards, and lack of privacy (Jakobsson et al., 2006; Tiernan et al., 2002). However, nearly half of the home alone clients in one study did not choose home as their preferred place of death (Aoun & Skett, 2013), prompting the authors to conclude that ‘the ability to die in the place of choice needs to be looked at as a possible indicator of meeting patient needs or as a quality measure in end of life care’ (p. 5).

### Implications for palliative care services

Community-based care is a vital component of palliative care. However, unless additional funds and resources are provided for community-based palliative care services, the demographic projections indicate that these services will face increasing challenges in servicing the needs of clients living alone and with no caregiver. Palliative care support needs for individuals living alone without a caregiver remain somewhat obscured because, until recently, those living home alone have been an invisible yet disadvantaged subgroup. While places of care and death are important outcome variables for examination, status of living and caregiver arrangements need to be more than of peripheral interest. Although studies have been conducted to assess the characteristics of clients who are receiving home-based palliative care, limited research has been conducted to specifically assess the needs of those who live alone and who do not have access to a primary caregiver, despite this group making up a significant percentage of the overall population under study. Studies highlight that the changing living arrangements of older people – such as the increase in living alone – has important implications for planning and provision of care and treatment for cancer sufferers (Grundy et al., 2004; Jakobsson et al., 2006); however, few suggested models of care addressed these implications. Given the aging populations, maintaining and extending the proportion of home deaths will likely result in more work for family doctors, district nurses, social services, and palliative home care teams (Higginson et al., 1999). Furthermore, it has been argued that general improvements in home care support may only help those who are already at an advantage (Grande, Addington-Hall, & Todd, 1998). Real progress may only be achieved by identifying the factors behind the disadvantage of certain groups (those living alone being one of them) and targeting interventions to address the disadvantages (Gao et al., 2013).

The ability of services to meet the needs of this group is further complicated by a lack of definitional clarity. In most of the reviewed studies, living alone status was inferred either by absence of spouse, or being single, or by being home alone but having caregivers. Nevertheless, in studies with a clearer definition, the wide variation in the degrees of ‘home aloneness’ created an impediment to evaluating the effectiveness of interventions using a randomized controlled trial (RCT) approach (Aoun et al., 2013) and this variation confounded the findings in terms of how much informal support each patient was getting over and above the implemented models of care that were randomly allocated. Therefore, the authors concluded: ‘there is a need to develop a scaling system of patient need, depending on the extent and frequency of informal support being provided by family and friends, before further trials are undertaken’ (p. 188). Rolls et al. (2011) highlighted the vast range of informal support and non-blood family upon whom older people living alone draw – friends, neighbors, volunteers, church members, and former unmarried partners and ex-in-laws to name a few. Given the complexity of the definitional criteria of this group, the RCT approach used in Aoun, O'Connor et al. (2013) was not considered appropriate for the ‘home alone’ palliative care population who would have been better supported by providing each participant with a personalized model of care that would have met their particular needs. This would have avoided offering a model of care that was inappropriate for the stage of their illness or unwanted by the patient in some cases (Aoun et al., 2013).

There is a small but growing body of literature dedicated to examining the service needs of palliative care patients who live alone without a caregiver focused on specific support services, end-of-life preferences in terms place of care and death, and interventions to address the disadvantages of being home alone at end of life. Two studies (Aoun et al., 2012, 2013) tested two models of care to enable this group to remain at home for as long as possible and provided some directions to inform service planning for this growing and challenging population group. There is a clear need for more studies to examine the effectiveness of informal support networks, interventions, and models of care. Adequate and timely services based on evidence will then lead to more care being delivered at home, a reduction in hospitalizations, a better quality of life, and a capacity to die at home if this is the patient's wish (Aoun et al., 2007).

### Limitations and future directions

Our purpose in this review was to provide a broader, comprehensive overview of the field of support needs at the end of life for those living alone at home, as opposed to a systematic review designed to weigh the evidence relating to a specific question. The comprehensive approach used to reviewing this literature optimized the rigor of the search processes and robustness of the conclusions. Most research in this field is difficult to grade using traditional levels of evidence for systematic reviews (Aoun & Kristjanson, 2005) as there are challenges that constrain the random selection of samples, and sample sizes tend to be relatively small (Aoun & Nekolaichuk, 2014). Furthermore, while international work was sought in the search for articles on this topic, a considerable proportion of the research generally, and the testing of the two models of care specifically, was conducted in Australia. Although the findings seem particularly pertinent to one location, they nevertheless are likely to have international application, and therefore there is a need for more international research on this under-examined topic.

## Conclusion

A significant proportion of older people in the developed countries will spend the last year of their life in poor health and with a considerable burden of palliative care needs due to social isolation, co-morbid conditions, and frailty. This has raised and continues to raise challenges for the provision and delivery of health and community services at the end of life. The growing population of people living alone and the reduced availability of informal caregivers together mediate the capacity to remain at home at end of life. This is the first literature review on the support needs of terminally ill people living alone at home and it has provided a comprehensive background for understanding current knowledge and highlighting the significance of potential research in the field.
